# Multilocus coalescent analyses reveal the demographic history and speciation patterns of mouse lemur sister species

**DOI:** 10.1186/1471-2148-14-57

**Published:** 2014-03-24

**Authors:** Christopher Blair, Kellie L Heckman, Amy L Russell, Anne D Yoder

**Affiliations:** 1 Department of Biology, Duke University, Box 90338, BioSci 130 Science Drive, Durham, NC 27708, USA; 2 Department of Ecology and Evolutionary Biology, Yale University, New Haven, CT 06520, USA; 3 Department of Biology, Grand Valley State University, Allendale, MI 49401, USA

**Keywords:** Coalescent methods, Modes of speciation, Historical demography, Lemur evolution, *Microcebus*, Multilocus, Peripatric speciation

## Abstract

**Background:**

Debate continues as to whether allopatric speciation or peripatric speciation through a founder effect is the predominant force driving evolution in vertebrates. The mouse lemurs of Madagascar are a system in which evolution has generated a large number of species over a relatively recent time frame. Here, we examine speciation patterns in a pair of sister species of mouse lemur, *Microcebus murinus* and *M. griseorufus*. These two species have ranges that are disparately proportioned in size, with *M. murinus* showing a much more extensive range that marginally overlaps that of *M. griseorufus*. Given that these two species are sister taxa, the asymmetric but overlapping geographic ranges are consistent with a model of peripatric speciation. To test this hypothesis, we analyze DNA sequence data from four molecular markers using coalescent methods. If the peripatric speciation model is supported, we predict substantially greater genetic diversity in *M. murinus*, relative to *M. griseorufus*. Further, we expect a larger effective population size in *M. murinus* and in the common ancestor of the two species than in *M. griseorufus*, with a concomitant decrease in gene tree/species tree incongruence in the latter and weak signs of demographic expansion in *M. murinus*.

**Results:**

Our results reject a model of peripatric divergence. Coalescent effective population size estimates were similar for both extant species and larger than that estimated for their most recent common ancestor. Gene tree results show similar levels of incomplete lineage sorting within species with respect to the species tree, and locus-specific estimates of genetic diversity are concordant for both species. Multilocus demographic analyses suggest range expansions for *M. murinus*, with this species also experiencing more recent population declines over the past 160 thousand years.

**Conclusions:**

Results suggest that speciation occurred in allopatry from a common ancestor narrowly distributed throughout southwest Madagascar, with subsequent range expansion for *M. murinus*. Population decline in *M. murinus* is likely related to patterns of climate change in Madagascar throughout the Pleistocene, potentially exacerbated by continual anthropogenic perturbation. Genome-level data are needed to quantify the role of niche specialization and adaptation in shaping the current ranges of these species.

## Background

In recent years, there has been growing interest in using genealogical tree structure to reconstruct the demographic and temporal context of diverging populations and species [1-4]. This tree-based approach permits the examination of lineages as they have diverged in the past using principles derived from coalescent theory, since ancestral polymorphisms that are shared among lineages are sorted during population segregation and speciation. A comparative demographic approach utilizing tree-based methods has been implemented in a variety of biological sub-fields, including systematics, phylogeography, conservation, and life history. Further, estimates of divergence times, migration rates, and effective population sizes among groups of organisms are now inferred with consideration of the genealogical structure of multilocus data sets [5-9]. These estimates may be synthesized and compared among sister taxa and used to infer the impact of the biogeographic or climatic context or other historical processes on patterns of genetic differences among groups [10-12].

In general, it is assumed that a complex suite of geologic, climatic, and population genetic forces have led to divergence and speciation in allopatry, where a reproductive barrier effectively divides an ancestral species into two populations of roughly equal size. With the continual progress of next-generation DNA sequencing and the increasing abundance of sequence data from across the genome, researchers are beginning to test alternate models of speciation including parapatric divergence along ecological clines [13-15]. Few studies, however, have used recently developed multilocus coalescent methods to fully understand the geography of speciation and the frequency of peripatric events in nature. Peripatric speciation can be seen as a subset of allopatric speciation, with a founder effect leading to the formation of a new species as a small population becomes physically separated from a broadly distributed ancestor. By definition then, the effective population size (*N*_e_) of the diverging population will be smaller than that of the common ancestor in a peripatric scenario. Although evidence of peripatric divergence is commonly inferred throughout archipelagos using traditional phylogenetic approaches (e.g. [16,17]), examples from strictly mainland taxa are relatively rare.

New algorithms can effectively estimate demographic parameters on a species tree to help disentangle the historical context of divergence and speciation and to test the plausibility of a peripatric scenario in comparison with the generally accepted null model of allopatric divergence [2,3,9]. Mitochondrial DNA (mtDNA) has been commonly used in studies of speciation given its small effective population size relative to nuclear DNA (nDNA) loci. Accordingly, mitochondrial gene trees undergo lineage sorting much more rapidly, and thus have a higher probability of yielding gene trees that are reciprocally monophyletic for the hypothesized species. However, the stochasticity inherent in single-locus gene trees makes this a controversial approach for estimating species histories, as previous authors have discussed (refer to [18-20]). Therefore, the implementation of a comparative demographic approach incorporating multiple unlinked loci is now regarded as a more powerful approach.

**Figure 1:**
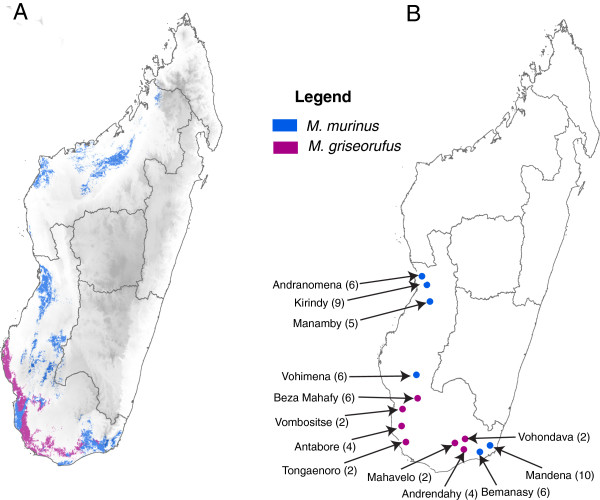
**Ranges and sampling information for all *Microcebus murinus *and *M. griseorufus *used in this study. A)** Map of Madagascar illustrating potential geographic ranges of *Microcebus murinus* and *M. griseorufus* based on species distribution modeling in Maxent. Ranges are visualized on a digital elevation model for Madagascar. Maxent models were performed by J.L. Brown and used with permission. **B)** Sampling information for individuals used in this study. Values in parentheses represent number of individuals included per locality.

Mouse lemurs (*Microcebus* spp.) are small, nocturnal primates that inhabit a wide range of habitats throughout Madagascar. As many as 21 species have been described within the genus [21-25], thus a remarkable increase from the two species taxonomy that was recognized as recently as 1994 [26,27]. The geography of the island has historically been considered to play a significant role in explaining divergence patterns among lemurs and other vertebrate species (e.g. [26,28-32]) and specifically *Microcebus* species [33-36]. Within the genus, however, *M. murinus* is unique in having a relatively vast geographic distribution. Whereas all other *Microcebus* species have geographically limited ranges --- some restricted to isolated forest fragments --- *M. murinus* is distributed along most of the western half and along the south of the island (Figure 1A). Over the extensive range of *M. murinus*, the species has been found to overlap with multiple congeners [37,38], many of which exhibit a high degree of endemicity. One example of this pattern of sympatry couples *M. murinus* with *M. griseorufus*, with multiple lines of evidence for distribution overlap and potential hybridization between the two species in southern Madagascar [39-41]. Within their respective ranges, *M. murinus* and *M. griseorufus* also exhibit marked differences in abundance. *Microcebus murinus* is a common, widespread species throughout the west (Figure 1A) that has been studied extensively since its description [26,27,42]. Conversely, *M. griseorufus* has been recorded from relatively few locations within a narrow range in the southwest of the island in the recent past [21,24].

**Figure 2:**
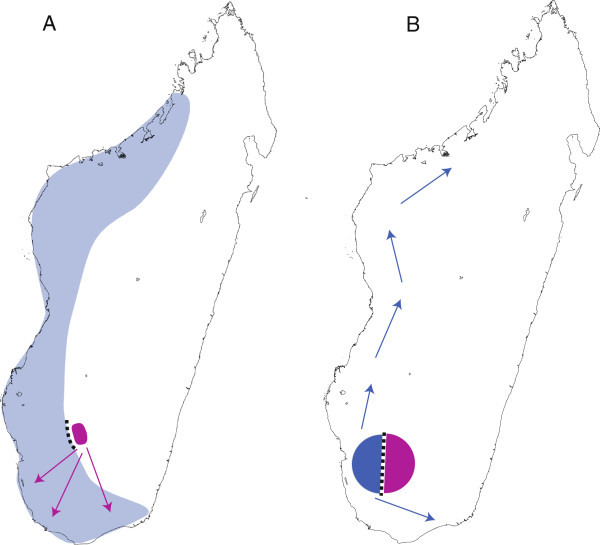
**Alternative speciation models. A)** Peripatric speciation model: a small founding population of *M. griseorufus* (purple) diverged from the widespread common ancestor (transparent blue) and then expanded in size. **B)** Allopatric speciation with subsequent range expansion model: a localized admixed common ancestor underwent divergence, after which *M. murinus* greatly expanded its range throughout the west (blue arrows), while expansion in *M. griseorufus* was relatively limited to the south.

Despite their partially overlapping distributions and large genetic distances [37], *M. murinus* and *M. griseorufus* are sister taxa [34,43,44]. Given the current ranges of *M. murinus* and *M. griseorufus* and their status as sister species, we consider two possible scenarios for the speciation event within this small clade. One scenario is that *M. griseorufus* was derived from the most recent common ancestor (MRCA) as a small founding population, with divergence the result of random genetic drift (Figure 2A). Alternatively, we consider that the MRCA was a small, narrowly distributed population located in southwestern Madagascar. Under this alternative hypothesis, the two modern species resulted from an event that partitioned that ancestral species into comparably sized populations prior to a substantial range expansion in *M. murinus* (Figure 2B). These two hypotheses differ most critically in their assumptions regarding the ancestral range and population size: was the MRCA more like modern *M. murinus* (hypothesis 1: peripatric speciation) or more like modern *M. griseorufus* (hypothesis 2: allopatric speciation)?

To test these alternative scenarios, we analyze multilocus DNA sequence data with coalescent-based methods to estimate relevant demographic parameters. If *M. griseorufus* diverged as a small founding population from a large ancestral population (Figure 2A), then we expect 1) that *M. murinus* has a greater effective population size and higher levels of genetic diversity than *M. griseorufus*, 2) that estimates of effective population size for the MRCA are more similar to those for *M. murinus* than to those for *M. griseorufus*, 3) that historical changes in population size in *M. murinus* were minimal, and finally, 4) that *M. griseorufus* is more likely to segregate as a clade in individual gene trees. Under the alternative hypothesis of recent allopatric speciation (Figure 2B), we expect that 1) that *M. murinus* and *M. griseorufus* have similar effective population sizes and levels of genetic diversity, 2) that *N*_e_ estimates for the MRCA are smaller than those for contemporary species or similar to *M. griseorufus*, 3) that significant population expansion has occurred in *M. murinus*, and finally, 4) that individual gene trees are more likely to show incomplete lineage sorting between the two species.

## Methods

### Data assembly

To estimate relevant demographic parameters for both species, we mined GenBank for all available high-quality sequences as both *M. murinus* and *M. griseorufus* have been used in a number of recent molecular studies (e.g. [41,44]). We sought to utilize genes with sufficient sequence variation to reliably estimate coalescent parameters. We also chose data sets with samples covering a large portion of the known geographic range of both species to assemble the most geographically complete data set possible (Figure 1B; Additional file 1: Table S1). Although recent studies have suggested that *M. murinus* may possibly contain at least three undocumented species, [43-45], we treat these populations as *M. murinus sensu lato* for the purpose of this study. The final data set consisted of the following nuclear loci: alpha enolase intron (ENOL: 916 bp), alpha fibrinogen intron (FIB: 608 bp), von Willebrand factor intron (VWF: 795 bp). Although the Adora 3 gene has been sequenced numerous times within *Microcebus*, levels of variation were too low to provide any meaningful information for this study. We also assembled and concatenated information from two mitochondrial loci including cytochrome *b* (cyt*b*) and cytochrome *c* oxidase subunit II (COII) for a total of 1,141 bp. The total number of sequences per locus was as follows: ENOL = 110 sequences; FIB = 124 sequences; VWF = 86 sequences; mtDNA = 55 concatenated sequences. All of the nuclear sequences represented phased haplotypes, which were included for all subsequent analyses. *Cheirogaleus major* was used as an outgroup for all phylogenetic and demographic analyses (see Additional file 1: Table S1). Although *Cheirogaleus* can be considered a relatively distant outgroup, we were interested in adding a temporal component to our analyses (see below) and sought to maintain consistency with taxon sampling across analyses. All sequence manipulation was performed in Geneious v. 6.1.5 [46]. Multiple sequence alignments were conducted using MAFFT v. 7.017 [47] within Geneious.

### Sequence diversity and effective population sizes

We first tested each marker for signs of recombination using the program RDP v. 4.0 [48]. Each marker was tested for recombination events using the GENECONV [49], MaxChi [50], and RDP methods [51]. Default settings were used for all analyses. Because no signs of recombination were detected, all subsequent analyses utilized the entire read of each locus. General sequence diversity statistics for each locus and species including number of haplotypes, haplotype diversity, number of segregating sites, and nucleotide diversity were calculated using DnaSP v. 5.10.1 [52].

Using the species tree topology ((*M. murinus*, *M. griseorufus*), *Cheirogaleus major*) prescribed by both nuclear and mitochondrial phylogenies [43,44,53], we used the program Bayesian Phylogenetics and Phylogeography (BPP v. 2.2; [9]) to estimate coalescent-scaled population sizes (θ = 4N_e_μ) and time of divergence (τ = μt). This method accommodates the species tree divergence patterns as well as gene tree lineage sorting processes. A gamma prior *G*(2,1000), with mean 2/1000 = 0.002 was used for the population size parameters (θ values). The age of the root in the species tree (τ_0_) was also assigned a gamma prior *G*(2,1000), while the other divergence time parameters were assigned the Dirichlet prior ([9]; Equation 2). Locus-specific mutation rates were allowed to vary, and we specified a heredity multiplier value *G*(4,5) to account for the combined mtDNA and nDNA data. We also performed analyses using larger priors for both τ_0_ and θ (*G*(2,100)) to evaluate the sensitivity of our results to the choice of prior. For this study, the parameters of interest were θ_M_, θ_G_, θ_MG_, and τ_MG_, where M = *M. murinus*, G = *M. griseorufus*, and MG = the MRCA of *M. murinus* and *M. griseorufus*. The default numbers of generations and sampling intervals were used for all analyses. Due to computational issues with the full data set, all BPP analyses were implemented on a reduced data set consisting of approximately 30 sequences per locus per *Microcebus* species and one to six *Cheirogaleus* sequences per locus. Sequences were randomly sampled to encompass a broad geographic range for each species. Previous simulation-based studies have shown that similar sample sizes can be sufficient to infer speciation processes [54]. Additional analyses on further reduced data sets (e.g. 10 sequences per locus per species) yielded similar results. All analyses were run at least twice to check for consistency among runs.

### Demographic expansion

To further test between the alternative speciation hypotheses, and to determine if the relatively large geographic range of *M. murinus* was due to population expansion following the split with *M. griseorufus*, we tested for signs of demographic change through time for both species. Under a peripatric speciation model, an ancestral species with a large range similar to that of modern *M. murinus* would have diverged into *M. murinus* and *M. griseorufus* populations, with the former experiencing little to no demographic change associated with the speciation event and the latter experiencing a significant population bottleneck event. Conversely, under the allopatric speciation model, a narrowly-distributed ancestral species would have diverged into *M. murinus* and *M. griseorufus* populations, with the former subsequently experiencing a significant population growth event and the latter experiencing a relatively smaller but possibly detectable demographic change after the speciation event.

To test for signs of demographic expansion or contraction we implemented multilocus Bayesian methods. Specifically, we constructed extended Bayesian skyline plots (EBSPs; [55]) for both species using BEAST v. 1.7.5 [56]. Studies have shown that doubling the number of independent loci can reduce error and 95% credible intervals in demographic reconstruction by √2 [55]. Thus, compared to single locus estimates, our multilocus data provided a powerful approach for estimating demographic trends in mouse lemurs. First, we used jModeltest v. 2.1.4 [57,58] to calculate model likelihood scores for each locus and to estimate optimal models using BIC (Table 1). Because mtDNA is inherited as a single linked unit, and to minimize the computational burden for BEAST, the concatenated mtDNA data were treated as a single partition. The three nuclear loci were each specified as a separate partition for model fitting. We tested the likelihood of 24 commonly used models in BEAST.

**Table 1:** Nucleotide substitution models selected for different data partitions using BIC

**Species**	**Locus**	**Model**
*M. griseorufus*	mtDNA	HKY
	alpha-enolase	F81 + G
	alpha fibrinogen	HKY + G
	von Willebrand factor	HKY + G
*M. murinus*	mtDNA	HKY + I
	alpha-enolase	HKY + I + G
	alpha fibrinogen	HKY + I
	von Willebrand factor	HKY + G
*BEAST	mtDNA	HKY + G
	alpha-enolase	HKY + G
	alpha fibrinogen	HKY + G
	von Willebrand factor	HKY + I + G

The first two blocks represent per locus models selected for extended Bayesian skyline plot analyses. ‘*BEAST’ represents a reduced data set encompassing both species and the outgroup (*Cheirogaleus major*).

We were also interested in adding a temporal component to the demographic analyses. Although there is no fossil record for lemurs, recent studies have utilized fossil information from more distantly related groups to date divergence times within the lemurs (e.g. [53,59]). Using multiple calibration points outside the clade and relaxed clock methods, these authors estimated the split between *Cheirogaleus* and *Microcebus* to be approximately 25 million years ago (Ma). We first used this information to estimate the substitution rate for each of the test loci in BEAST using a reduced data set of both species and *Cheirogaleus* (see Additional file 1: Table S1). Best-fitting models were calculated and used for all divergence dating and rate estimation. Because alignments contained representative alleles from both multiple species and multiple individuals within species, we used *BEAST [60] to estimate the posterior distribution of substitution rates. We defined three species (*Cheirogaleus major*, *M. griseorufus*, *M. murinus*) and grouped alleles accordingly. For each analysis, the root node of the species tree was calibrated with a normal distribution around a mean of 25 Ma and standard deviation of 5 Ma, which encompassed the 95% HPD estimates from previous studies [51]. To increase computational efficiency, we ran four independent *BEAST analyses (by locus) to estimate the posterior distribution of rates. Both strict and relaxed clock (lognormal; [61]) models were tested. All analyses were run between 10–50 million generations; sampling was chosen at intervals to utilize 10,000 draws from the posterior. Following analyses, the program Tracer v1.4 [62] was used to examine effective sample size (ESS) values (target > 200) and examine the posterior distribution of relevant parameters. We used the 95% HPD of the substitution rate for each locus as a uniform prior to add a temporal component to all EBSP analyses. All EBSP analyses used a strict clock. Operators were modified according to author recommendations and analyses were run for 50 million generations (*M. griseorufus*) or 200 million generations (*M. murinus*) to obtain adequate ESS values. All EBSP and *BEAST analyses were implemented via the Duke Shared Cluster Resource (DSCR).

### Phylogenetic analysis

Under a scenario of peripatric speciation, we expected to detect differing signals in the degree of incomplete lineage sorting in gene trees for *M. griseorufus* and *M. murinus.* For example, some coalescence times within *M. murinus* would predate speciation, whereas *M. griseorufus* would show a higher degree of reciprocal monophyly among different loci due to its smaller effective population size, particularly during the bottleneck that was hypothesized to be associated with the peripatric speciation event. Conversely, under an allopatric speciation model we would expect the degree of incomplete lineage sorting among gene trees to be similar for both species. Thus, we performed maximum likelihood (ML) phylogenetic analysis of each locus using RAxML v. 7.6.0 [63]. Because all mitochondrial genes are linked, we performed a single ML analysis for the concatenated mtDNA loci. For each gene we ran a full ML analysis followed by rapid bootstrapping [64] using the *autoMRE* bootstopping criterion.

## Results

### Sequence diversity and effective population sizes

**Figure 3:**
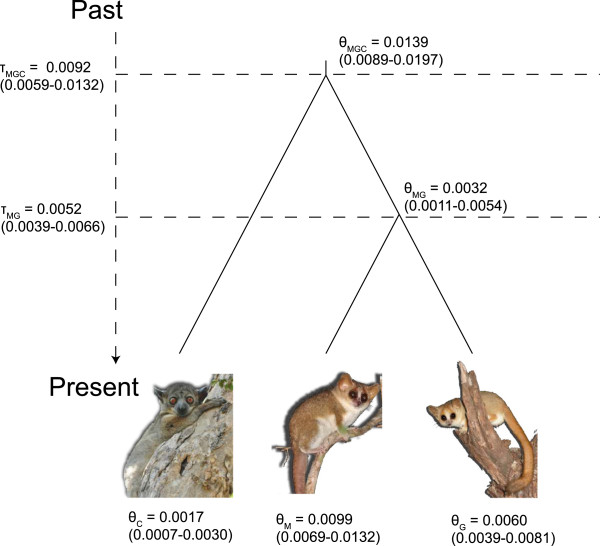
**BPP results illustrating effective population sizes (θ = 4*N***_**e**_**μ) and time of divergence (τ = μt).** Results are based on gamma priors *G*(2,1000) for θ and τ_0_. Values in parentheses represent 95% HPDs. C = *Cheirogaleus major*; M = *Microcebus murinus*; G = *Microcebus griseorufus*; MG = most recent common ancestor (MRCA) of *M. murinus* + *M. griseorufus*; MGC = MRCA of all the taxa. Photos of *Cheirogaleus* and *M. murinus* courtesy of J. L. Brown. Photo of *M. griseorufus* courtesy of K. Dausmann.

In general, sequence diversity characteristics showed moderate values for each gene for both species (Table 2). Although average values were slightly higher, *M. murinus* did not exhibit consistently higher diversity values than *M. griseorufus* based on haplotype diversity, nucleotide diversity, or average number of nucleotide differences*.* Multiple runs of BPP gave similar results indicating adequate sampling of the posterior. ESS values were also high for all parameters (Additional file 2: Table S2, Additional file 3: Table S3). Using the prior *G*(2,1000) for θ and τ_0_, mean effective population size was slightly greater for *M. murinus* (θ_M_ = 0.0099) versus *M. griseorufus* (θ_G_ = 0.0060). However, 95% HPDs for the two species almost completely overlapped (Figure 3). The MRCA had a significantly smaller mean population size than either species (θ_MG_ = 0.0032; 95% HPD 0.0011–0.0054). BPP results using a gamma prior of *G*(2,100) for θ and τ_0_ resulted in slightly larger parameter estimates (Additional file 3: Table S3). With these priors, the mean effective population size of the MRCA was intermediate between *M. murinus* and *M. griseorufus*. (θ_MG_ = 0.0208; 95% HPD 0.0087–0.0351; θ_M_ = 0.0316; 95% HPD 0.0183–0.0470; θ_G_ = 0.0185; 95% HPD 0.01026–0.02789). However, confidence intervals again overlapped substantially. In all analyses, effective population size estimates of the MRCA were more similar to *M. griseorufus* than they were to *M. murinus*.

**Table 2:** **Nucleotide diversity statistics for *Microcebus griseorufus *and *M. murinus***

**Locus**	**Species**	**n**	***S***	***h***	***Hd***	***pi***	***k***
mtDNA	*griseorufus*	22	41	14	0.948	0.00516	9.407
	*murinus*	33	75	17	0.909	0.01687	19.216
Alpha enolase	*griseorufus*	42	19	22	0.951	0.00713	6.051
	*murinus*	68	32	35	0.921	0.00939	6.848
Alpha fibrinogen	*griseorufus*	44	13	9	0.68	0.00822	4.933
	*murinus*	80	36	20	0.799	0.00842	5.046
von Willebrand factor	*griseorufus*	26	20	15	0.926	0.01072	5.862
	*murinus*	60	39	27	0.955	0.00806	6.024
Average	*griseorufus*	33.5	23.25	15	0.876	0.00781	6.563
	*murinus*	60.25	45.5	24.75	0.896	0.0107	9.284

### Demographic expansion

**Figure 4:**
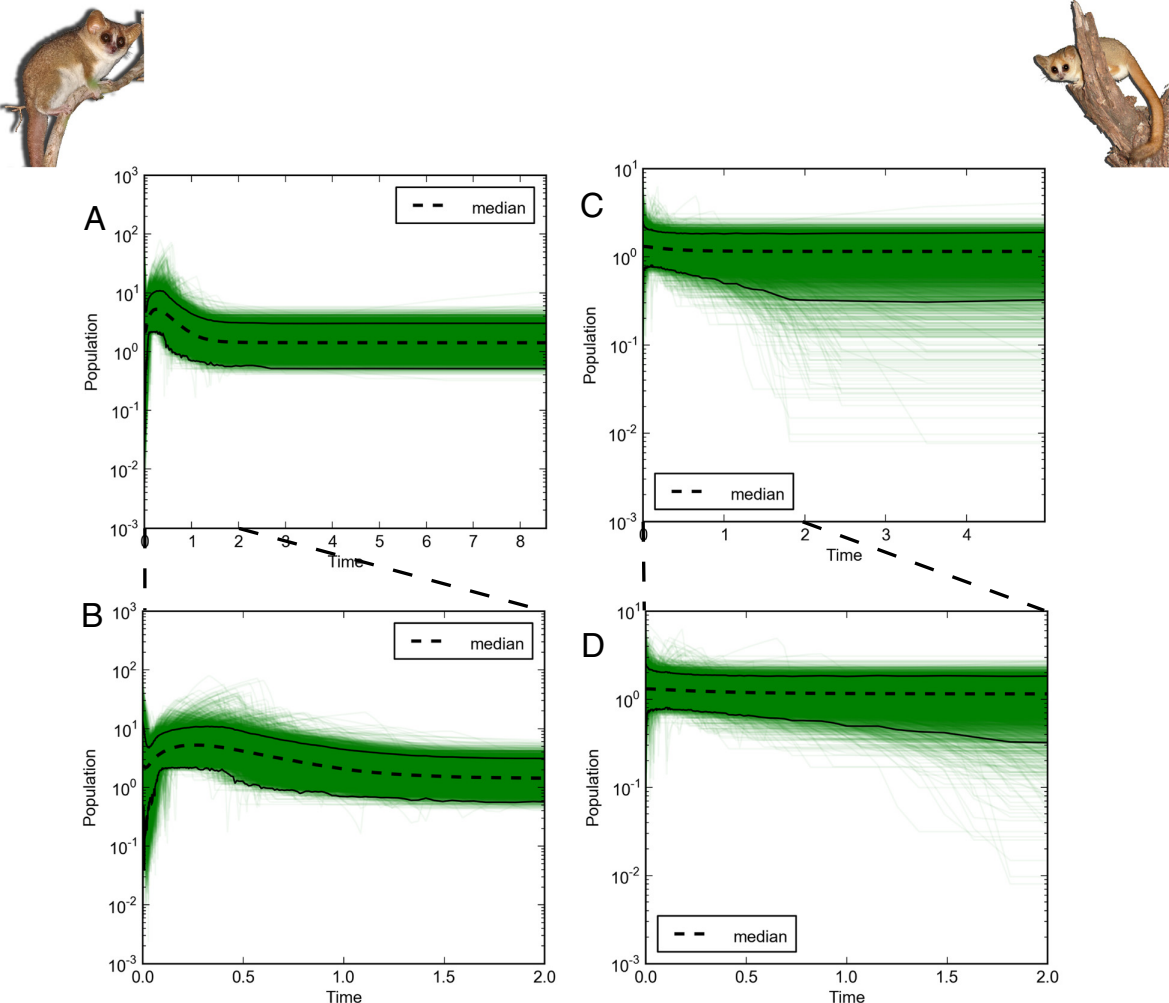
**Extended Bayesian skyline plots illustrating the entire posterior distribution of demographic trends for *Microcebus murinus *(A,B) and *M. griseorufus *(C,D).** Top panels represent demographic trends from the MRCA of the respective species based on divergence times from the oldest gene, whereas bottom panels represent more recent population size changes through the Pleistocene. Dotted lines indicate median effective population size whereas solid lines represent 95% HPDs. Time is in units of millions of years before present. Population sizes are in log units.

For all *BEAST analyses, the 95% HPD for the coefficient of variation parameter included zero for all relaxed clock analyses, indicating that a strict clock was sufficient to explain the data. Mean estimated rates of nucleotide substitution (substitutions per site per million years) and 95% HPDs for each locus were as follows: mtDNA = 0.0132 [0.0060862–0.0222]; ENOL = 0.0025731 [0.00037366–0.0046715]; FIB = 0.0010495 [0.00027744–0.0019127]; VWF = 0.0023935 [0.00037916–0.0045323]). EBSP results for *M. murinus* indicated an increase in effective population size at about 1 Ma, with a subsequent rapid decline in size starting approximately 160 Ka and continuing to the present (Figure 4A,B). The mean number of population size changes throughout the history of *M. murinus* was estimated as 2.4 (95% HPD 2–4). Conversely, results for *M. griseorufus* showed signs of relatively constant population size through time (Figure 4C,D) with an estimated mean of 0.59 size changes (95% HPD 0–2).

### Phylogenetic analysis

Maximum likelihood phylogenetic analyses of the mtDNA data revealed reciprocal monophyly for both species with strong bootstrap support (Additional file 4: Figure S1). Gene copies within *Microcebus griseorufus* were also monophyletic based on ML analysis of the ENOL locus (bootstrap support = 81; Additional file 5: Figure S2). Conversely, none of the nuclear markers showed reciprocal monophyly for *M. murinus*. Moderate geographic structure among populations was detected from the mtDNA analysis, particularly for *M. murinus* (Additional file 4: Figure S1). However, nuclear gene tree analyses suggested little to no signal of geographic population structure within species as haplotypes were shared among many localities (Additional file 5: Figure S2, Additional file 6: Figure S3, Additional file 7: Figure S4). Species tree analyses suggested that divergence of *M. griseorufus* and *M. murinus* occurred approximately 3–6 Ma.

## Discussion

### Speciation models

Our motivation for this study was to differentiate between competing models of speciation that can potentially explain the historical divergence between a sister species pair of mouse lemurs. These primates are of particular interest for such a study given their cryptic species diversity, highly threatened status, and their endemic distribution in Madagascar, one of Earth’s hottest biodiversity hotspots. Given the difference in the sizes of their geographic distributions (Figure 1A), we initially hypothesized that *M. griseorufus* diverged peripatrically from a geographically widespread common ancestor (Figure 2A)*.* The observation that two sister species occur with different yet overlapping ranges in Madagascar raises obvious questions regarding the driving mechanisms behind their divergence. Given that one species, *M. griseorufus*, shows a more limited though contiguous range with *M. murinus* is on its face entirely congruent with a peripatric model of speciation. To test this hypothesis, we formed a set of predictions that rely on a series of historical demographic variables including comparisons of effective population size in both the modern species and their common ancestor, as well as patterns of population size change in the history of the focal species. The majority of our results reject a model of peripatric speciation in favor of a model of allopatric divergence with subsequent range expansion for *M. murinus*.

### Contemporary and ancestral N_e_

Our BPP results using both large and small priors for divergence times and population sizes strongly suggest that contemporary *N*_e_ values are similar for both species. For example, although mean estimates of *N*_e_ for *M. murinus* are slightly larger than those for *M. griseorufus*, 95% HPDs overlapped significantly. In analyses using the priors *G*(2,1000), *N*_e_ estimates of the MRCA are substantially smaller than for either contemporary species, although using larger priors results in *N*_e_ estimates of the MRCA that are intermediate to the two contemporary species. However, regardless of the prior used, *N*_e_ estimates for the MRCA were more similar to *M. griseorufus* than to the more widespread *M. murinus*. These results support a model in which the MRCA was a species with a relatively small geographic distribution likely situated within southern Madagascar. These conclusions are congruent with our phylogenetic results and with estimates of genetic diversity for both species. Although genetic diversity is slightly higher in *M. murinus*, this may be an artifact of both our sampling regime and/or recent demographic trends for this species (see below). Under a peripatric scenario we would expect to find substantially larger diversity values in *M. murinus*, a pattern that was not recovered with any marker. The reciprocal monophyly and population structure we observed with the mtDNA and incomplete lineage sorting in nuclear markers is also congruent with other recent studies of these species (e.g. [43,44]).

These results strongly support the allopatric rather than the peripatric speciation model. Of particular importance for this conclusion is the estimation of *N*_e_ for the MRCA of *M. murinus* and *M. griseorufus*. The model employed in BPP allows for the combined analysis of multiple genetic markers in a coalescent framework, a necessary approach as individual loci may suffer from rate heterogeneities and idiosyncratic gene genealogies [65,66]. Because a single genetic locus provides a limited and highly stochastic perspective, we find that examining four independent loci is effective for estimates when combined, but may remain insufficient for reconciling historical demographic processes when analyzed individually given the limited power of single locus analyses [5,53]. A rejection of a peripatric model for mouse lemurs is also similar to a recent study on mantellid frogs based on range overlap analysis, where the authors found that range size differences among sister species increased with evolutionary age [67]. For example, many sister species of frogs were composed of microendemics encompassing similarly-sized geographic ranges. Under a peripatric scenario, range asymmetry would be high in younger species.

### Demographic changes

Our results regarding population size changes provide further support for the allopatric model. Although only four independent loci are used to infer demographic changes, four loci are predicted to reduce the error by one-half as compared to single-locus estimates [55]. EBSP results suggest demographic expansion for *M. murinus* in the Quaternary around 1 Ma. Demographic expansion during the Quaternary has also been documented previously for populations of *M. murinus* in northwest Madagascar [68] as well as for Malagasy rodents [69]. However, our results differ from previous single-locus studies of *M. murinus* that have utilized different analytical methods and found evidence for more recent episodes of expansion during the Pleistocene and Holocene [68]. Evidence from palynological records indicates that the Pleistocene climate and vegetation of Madagascar, like most of the world, was quite different than the climate of today [70]. For example, during the Last Glacial Maximum (LGM) ~40–20,000 years ago, humid forest was likely restricted to isolated refugia scattered throughout the island, whereas dry, xeric vegetation was allowed to expand. Indeed, a wide body of evidence is available that suggests that a large portion of Madagascar experienced substantially drier conditions during the LGM than the present (see [70]). Both *M. murinus* and *M. griseorufus* are common in dry environments and both were likely affected by climate conditions associated with Quaternary Madagascar.

Following the demographic expansion of *M. murinus* during the Quaternary, our results suggest recent and substantial population decline of this species beginning approximately 160 Ka and continuing to the present. It has been proposed that vegetation shifts associated with Pleistocene climate change were more substantial in western dry forest versus the arid spiny forest to the south [68], which may partly explain why no evidence of recent population decline is indicated for *M. griseorufus*. Furthermore, evidence suggests that humans first colonized Madagascar ~2,000 years ago and subsequently had a rapid and profound impact on the native biota and their habitats [70,71]. A variety of hypotheses have been put forth to explain the decline of Malagasy flora and fauna subsequent to human colonization, including increased frequency of fire [72], drought [73], hunting [74], invasive species [75], disease [70], and synergistic anthropogenic influences [76]. Regardless of the exact mechanism(s), it is highly probable that the recent and rapid population decline inferred from our data for *M. murinus* has been exacerbated by subsequent anthropogenic influences beginning around 2,000 years ago. The relatively constant population size of *M. griseorufus* suggests that human impacts and habitat fragmentation throughout the southern spiny forests may have been less severe than impacts throughout western dry forests.

### Alternative speciation hypotheses

Our results indicate that the large geographic range of *M. murinus* seems to be a uniquely derived feature of this species. This phenomenon begs investigation. Even so, the evidence presented in this study fails to specifically explain the mechanism either promoting the geographic range expansion of *M. murinus* or limiting the range of *M. griseorufus*. The initial divergence between the two species may have resulted from a geographically-based vicariant event or an ecological niche separation. There is no discernible extrinsic barrier separating the two species, but there is evidence that suggests ecological segregation [39-41,77,78]. Although some studies suggest that *M. murinus* preferentially inhabits dry forest habitat in northwestern Madagascar [79], it could be argued that *M. murinus* is a generalist species as it is often found in both dry deciduous forest and wet, gallery forest habitats in the southeast [38,39]. These habitat associations contrast with *M. griseorufus*, which is more common in xeric, spiny forest [21,39], though recently *M. griseorufus* has been shown to inhabit both spiny and gallery forests at the Beza Mahafaly Private Reserve [45]. Therefore, while these habitat preferences may have been important in the original subdivision of the ancestral species, there is no direct evidence supporting the hypothesis that this drove their initial divergence. The use of next-generation DNA sequencing methods, in particular RAD-Seq or whole transcriptomes, may be useful for investigating and quantifying genomic islands of divergence and adaptation in this system. Additionally, multilocus demographic methods such as those used here should be combined with future projections of species distribution models to better inform conservation practices. This is of utmost importance for highly threatened taxa inhabiting areas that are experiencing high rates of habitat loss, such as the case with Madagascar’s lemur fauna [80,81].

## Conclusions

We estimated historical demographic parameters in a multilocus coalescent framework to test the predictions associated with two models of speciation that may have driven the divergence of *M. griseorufus* and *M. murinus*. The majority of our results reject the hypothesis of peripatric speciation. Our results instead favor a model of allopatric divergence from a range-restricted common ancestor in southwestern Madagascar, with subsequent range expansions for *M. murinus*. Whether due to ecological constraint or interspecific competition, *M. griseorufus* is presently restricted to the arid spiny forest in the south, whereas *M. murinus* has successfully expanded throughout much of western Madagascar and limited areas to the southeast. The methods used here can be easily applied to address similar evolutionary questions in other systems to help elucidate the geographic context of divergence and speciation. In turn, these approaches can help guide conservation priorities when synthesized with complex geospatial methods and species distribution models.

## Abbreviations

Ne: Effective population size; mtDNA: mitochondrial DNA; nDNA: nuclear DNA; MRCA: Most recent common ancestor; ENOL: Alpha enolase intron; FIB: Alpha fibrinogen intron; VWF: Von Willebrand factor intron; cytb: cytochrome *b*; COII: Cytochrome *c* oxidase subunit II; BPP: Bayesian Phylogenetics and Phylogeography; Ma: Million years ago; ESS: Effective sample size; DSCR: Duke Shared Cluster Resource; ML: Maximum likelihood; HPD: Highest posterior density; θM: Effective population size of *Microcebus murinus*; θG: Effective population size of *Microcebus griseorufus*; θMG: Effective population size of the most recent common ancestor; τMG: Divergence time of the most recent common ancestor; Ka: Thousand years ago; LGM: Last Glacial Maximum.

## Competing interests

The authors declare that they have no competing interests.

## Authors’ contributions

CB, KH, AR, ADY designed the study. CB collected and analyzed the data. CB, AR, ADY wrote the paper. All authors read an approved the final manuscript.

## Authors’ information

CB is presently a postdoctoral associate in the laboratory of Anne Yoder at Duke University. His research interests include combining next-generation DNA sequencing data with spatially explicit modeling to understand the contemporary and historical processes responsible for shaping patterns of genomic variation in natural populations. He is also interested in using next-generation sequencing of environmental samples to quantify patterns of biodiversity.

## Supplementary Material

Additional file 1: Table S1 Database of all individuals and haplotypes used for this study. The last four columns indicate which haplotypes were used for each analysis. Click here for file

Additional file 2: Table S2 Results from BPP analysis using priors *G*(2,1000) for both τ0 and θ. Parameters were estimated on a fixed species tree (*Cheirogaleus major* (*M. murinus*, *M. griseorufus*)). Click here for file

Additional file 3: Table S3 Results from BPP analysis using priors *G*(2,100) for both τ0 and θ. Parameters were estimated on a fixed species tree (*Cheirogaleus major* (*M. murinus*, *M. griseorufus*)). Click here for file

Additional file 4: Figure S1 Maximum likelihood mtDNA gene tree (concatenated cytochrome *b* and cytochrome *c* oxidase II) for all *Microcebus griseorufus* (purple) and *M. murinus* (blue) sequences used for this study. Values at nodes represent bootstrap support values >50 calculated using the *autoMRE* function in RAxML. For ease of visualization the outgroup taxon (*Cheirogaleus major*) is removed. Click here for file

Additional file 5: Figure S2 Maximum likelihood alpha enolase gene tree for all *Microcebus griseorufus* (purple) and *M. murinus* (blue) sequences used for this study. Values at nodes represent bootstrap support values >50 calculated using the *autoMRE* function in RAxML. For ease of visualization the outgroup taxon (*Cheirogaleus major*) is removed. Click here for file

Additional file 6: Figure S3 Maximum likelihood alpha fibrinogen gene tree for all *Microcebus griseorufus* (purple) and *M. murinus* (blue) sequences used for this study. Values at nodes represent bootstrap support values >50 calculated using the *autoMRE* function in RAxML. For ease of visualization the outgroup taxon (*Cheirogaleus major*) is removed. Click here for file

Additional file 7: Figure S4 Maximum likelihood von Willebrand factor gene tree for all *Microcebus griseorufus* (purple) and *M. murinus* (blue) sequences used for this study. Values at nodes represent bootstrap support values >50 calculated using the *autoMRE* function in RAxML. For ease of visualization the outgroup taxon (*Cheirogaleus major*) is removed. Click here for file
