# Supplementary figures and images for: Multilocus coalescent analyses reveal the demographic history and speciation patterns of mouse lemur sister species
Source: BMC Evol Biol. 2014 Mar 24;14:57. doi: 10.1186/1471-2148-14-57 (PMC3987692; doi:10.1186/1471-2148-14-57)

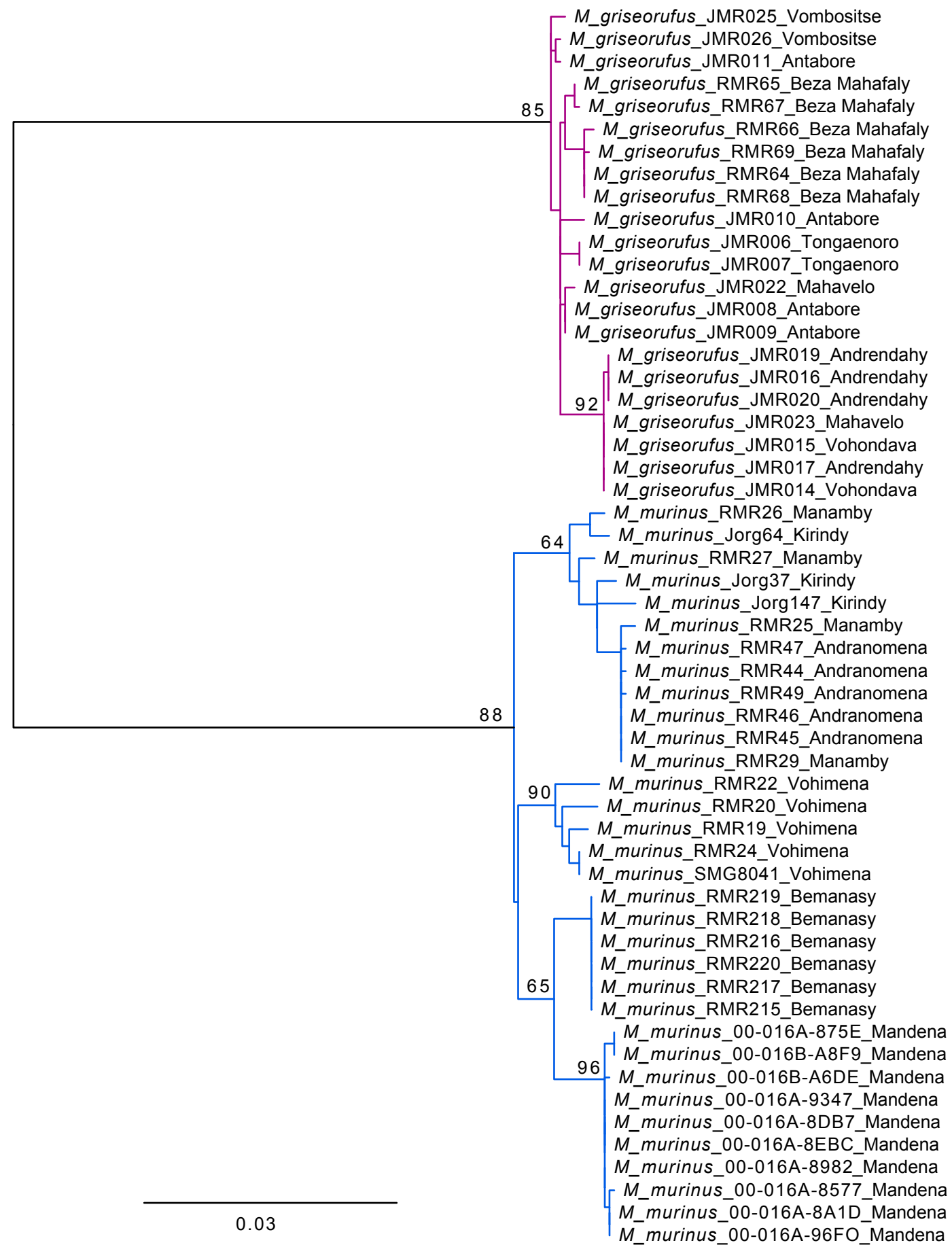

Supplement: Additional file 4: Figure S1 — Maximum likelihood mtDNA gene tree (concatenated cytochrome b and cytochrome c oxidase II) for all Microcebus griseorufus (purple) and M. murinus (blue) sequences used for this study. Values at nodes represent bootstrap support values >50 calculated using the autoMRE function in RAxML. For ease of visualization the outgroup taxon (Cheirogaleus major) is removed. [file 1471-2148-14-57-S4.pdf]

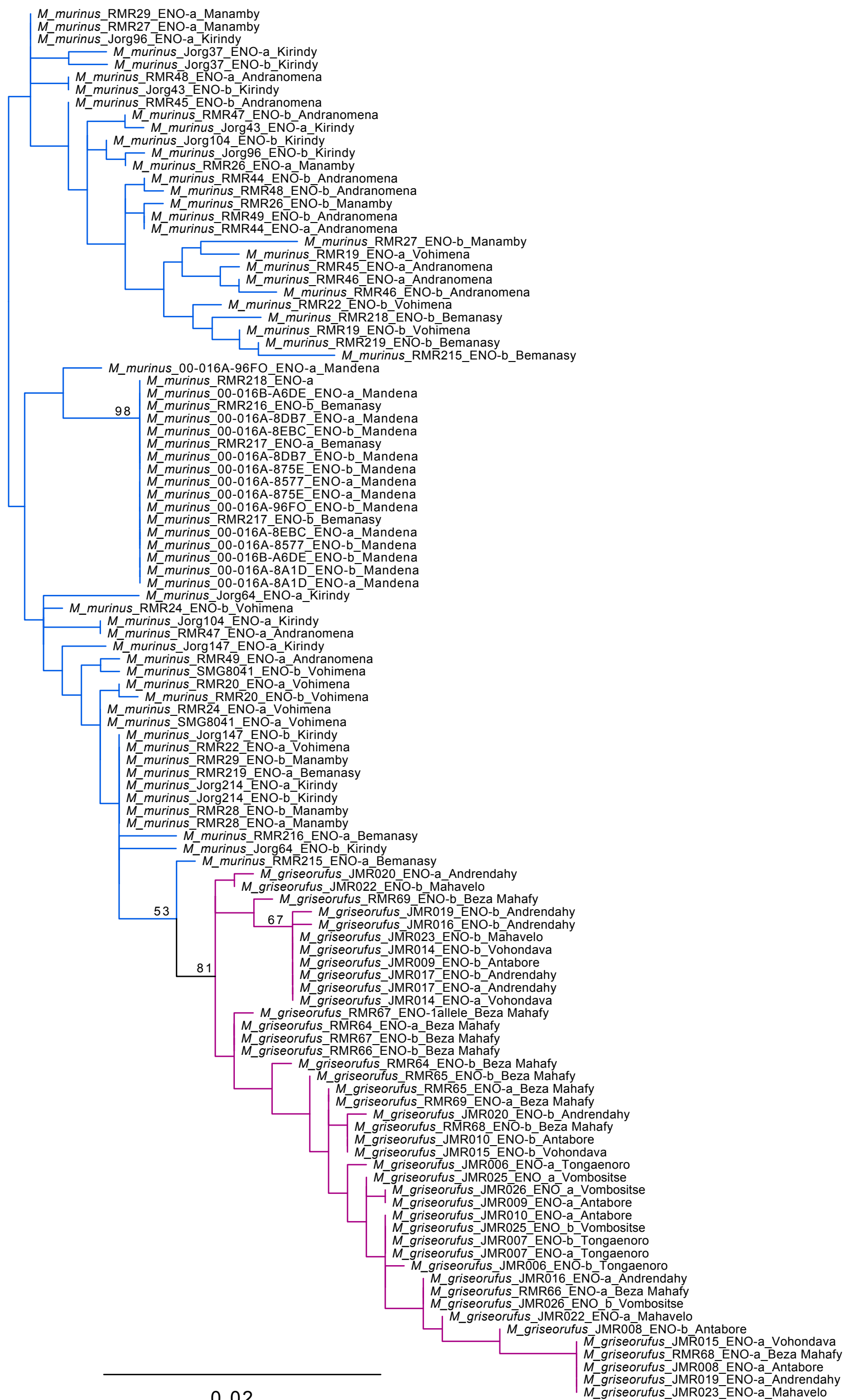

0.02

Supplement: Additional file 5: Figure S2 — Maximum likelihood alpha enolase gene tree for all Microcebus griseorufus (purple) and M. murinus (blue) sequences used for this study. Values at nodes represent bootstrap support values >50 calculated using the autoMRE function in RAxML. For ease of visualization the outgroup taxon (Cheirogaleus major) is removed. [file 1471-2148-14-57-S5.pdf]

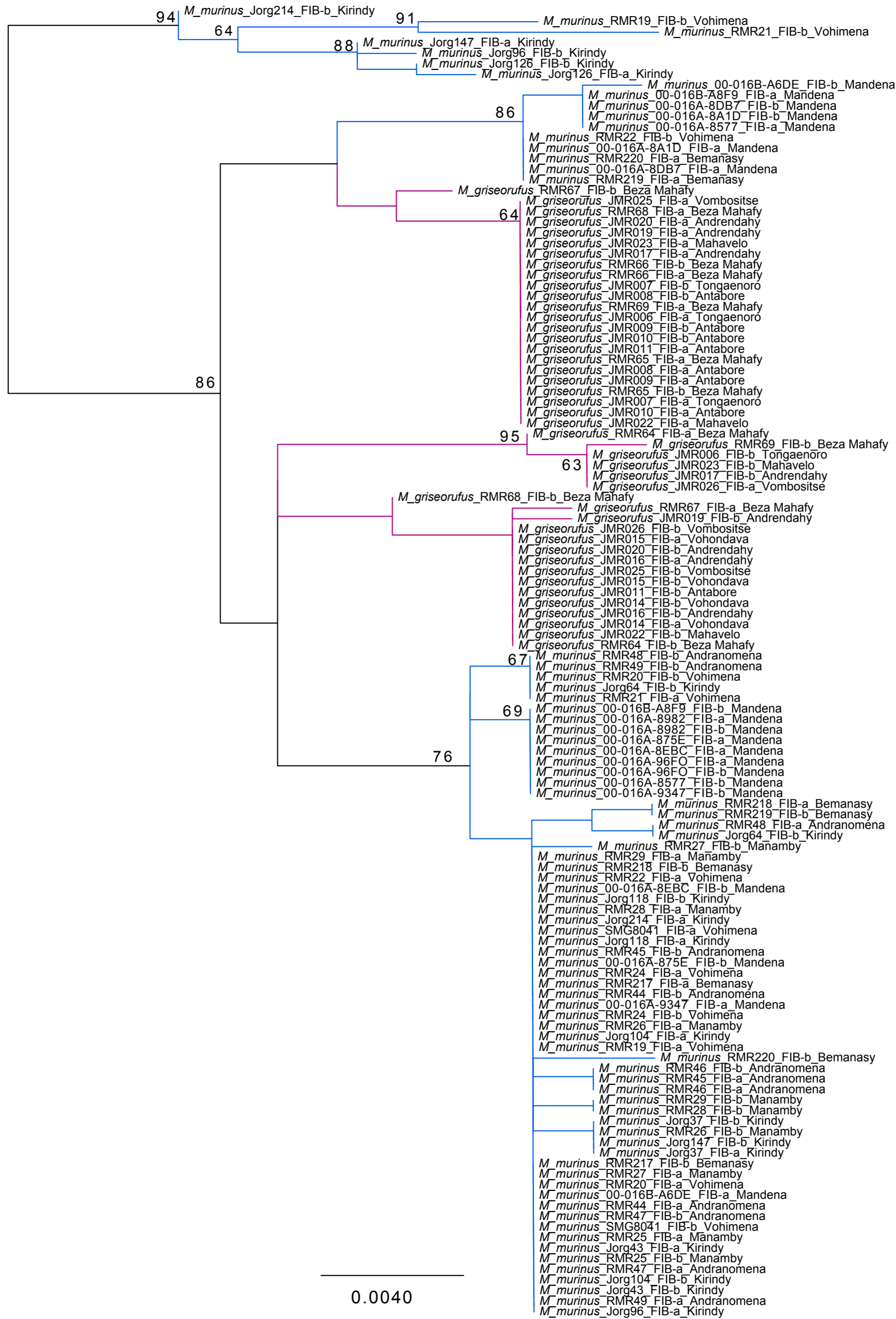

Supplement: Additional file 6: Figure S3 — Maximum likelihood alpha fibrinogen gene tree for all Microcebus griseorufus (purple) and M. murinus (blue) sequences used for this study. Values at nodes represent bootstrap support values >50 calculated using the autoMRE function in RAxML. For ease of visualization the outgroup taxon (Cheirogaleus major) is removed. [file 1471-2148-14-57-S6.pdf]

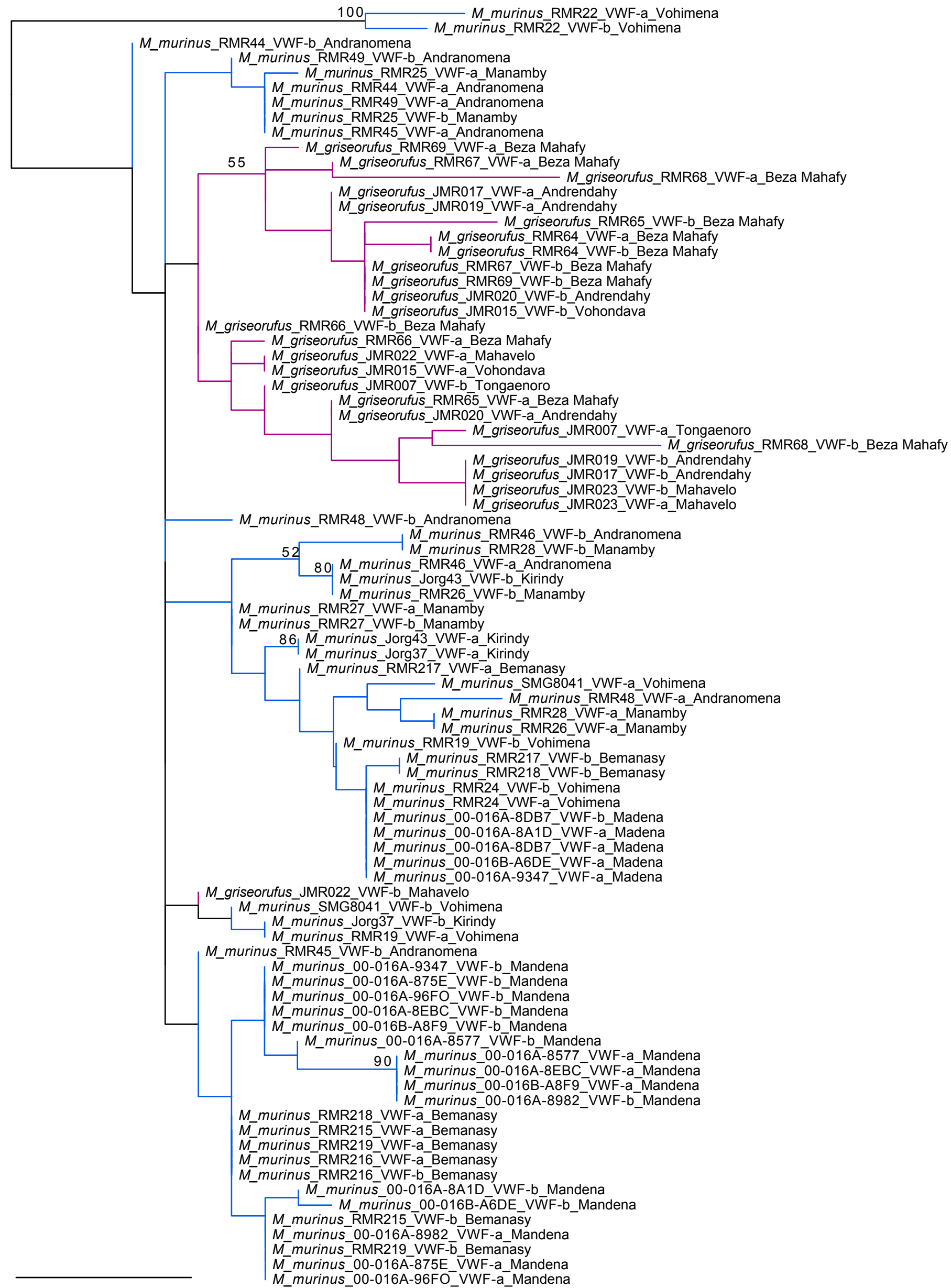

0.0080

Supplement: Additional file 7: Figure S4 — Maximum likelihood von Willebrand factor gene tree for all Microcebus griseorufus (purple) and M. murinus (blue) sequences used for this study. Values at nodes represent bootstrap support values >50 calculated using the autoMRE function in RAxML. For ease of visualization the outgroup taxon (Cheirogaleus major) is removed. [file 1471-2148-14-57-S7.pdf]
